# Chondroitin Sulfate Proteoglycans Revisited: Its Mechanism of Generation and Action for Spinal Cord Injury

**DOI:** 10.14336/AD.2023.0512

**Published:** 2024-02-01

**Authors:** Rui Yang, Ying Zhang, Jianning Kang, Ce Zhang, Bin Ning

**Affiliations:** ^1^Jinan Central Hospital, Shandong University, Jinan, Shandong, China.; ^2^Central Hospital Affiliated to Shandong First Medical University, Shandong First Medical University & Shandong Academy of Medical Sciences, Jinan, Shandong, China

**Keywords:** CSPGs, Extracellular traps, Inflammation, Reactive astrocytes, Spinal cord injury

## Abstract

Reactive astrocytes (RAs) produce chondroitin sulfate proteoglycans (CSPGs) in large quantities after spinal cord injury (SCI) and inhibit axon regeneration through the Rho-associated protein kinase (ROCK) pathway. However, the mechanism of producing CSPGs by RAs and their roles in other aspects are often overlooked. In recent years, novel generation mechanisms and functions of CSPGs have gradually emerged. Extracellular traps (ETs), a new recently discovered phenomenon in SCI, can promote secondary injury. ETs are released by neutrophils and microglia, which activate astrocytes to produce CSPGs after SCI. CSPGs inhibit axon regeneration and play an important role in regulating inflammation as well as cell migration and differentiation; some of these regulations are beneficial. The current review summarized the process of ET-activated RAs to generate CSPGs at the cellular signaling pathway level. Moreover, the roles of CSPGs in inhibiting axon regeneration, regulating inflammation, and regulating cell migration and differentiation were discussed. Finally, based on the above process, novel potential therapeutic targets were proposed to eliminate the adverse effects of CSPGs.

## 1. Introduction

Spinal cord injury (SCI), a serious injury of the central nervous system (CNS), is followed by an unrecoverable movement disorder, sexual dysfunction and defecation disorder [1, 2]. The primary damages, including ischemia, hypoxia, and oxidative stress, are caused by SCI, and further lead to secondary damage [3]. In the secondary injury, the cells and the extracellular matrix (ECM) undergo various changes, which eventually cause the formation of scar tissue to wrap the lesion core and limit axon regeneration, thereby leading to the failure of functional recovery [2]. Among the ECMs, chondroitin sulfate proteoglycans (CSPGs) are the most prominent ECMs, which inhibit axonal regeneration [2, 4]. Therefore, the generation and action mechanisms of CSPGs for the recovery of SCI need to be studied. Recent studies have found that neutrophils and microglia produce and release extracellular traps (ETs), which are reticular extracellular fibers in SCI [5]. They can aggravate the secondary injury and stimulate the activation of astrocytes to produce CSPGs [6-10]. Moreover, reactive astrocytes (RAs) can induce the overexpression of CSPGs through various intracellular signal transduction processes, which eventually leads to the formation of numerous CSPGs in SCI [11-14].

There are several types of CSPGs, including CSPG1 (Aggrecan), CSPG2 (Versican), CSPG3 (Neurocan), CSPG4 (NG2), CSPG7 (Brevican), etc. [15]. Among them, Aggrecan, Versican, Neurocan, and Brevican belong to the lectican family and form perineuronal nets (PNNs), which inhibit axon regeneration [2, 16]. The CSPGs reviewed in the current article are referred to as lectican-like CSPGs. The CSPGs form PNNs around neurons and inhibit the regeneration of axons [2]. The CSPGs inhibit axon regeneration by acting on different receptors, and their downstream pathways converge in the Rho/ Rho-associated protein kinase (ROCK) pathway [2, 4]; however, there are some differences. Moreover, the CSPGs regulate inflammation with different or even opposite effects at different SCI time points [17, 18]. There are also differences in the regulation of migration and differentiation of different cells [19, 20].

Given the above CSPGs formation and action mechanism, the current paper reviewed therapeutic strategies, which target five targets, including histone deacetylase 6 (HDAC6), ETs, RAs, receptors and enzymes. Furthermore, the research direction and treatment strategies based on the mechanism of CSPGs after SCI as well as the future development prospects were also discussed.

## 2. Generation of CSPGs: ETs and RAs

**Figure 1.**
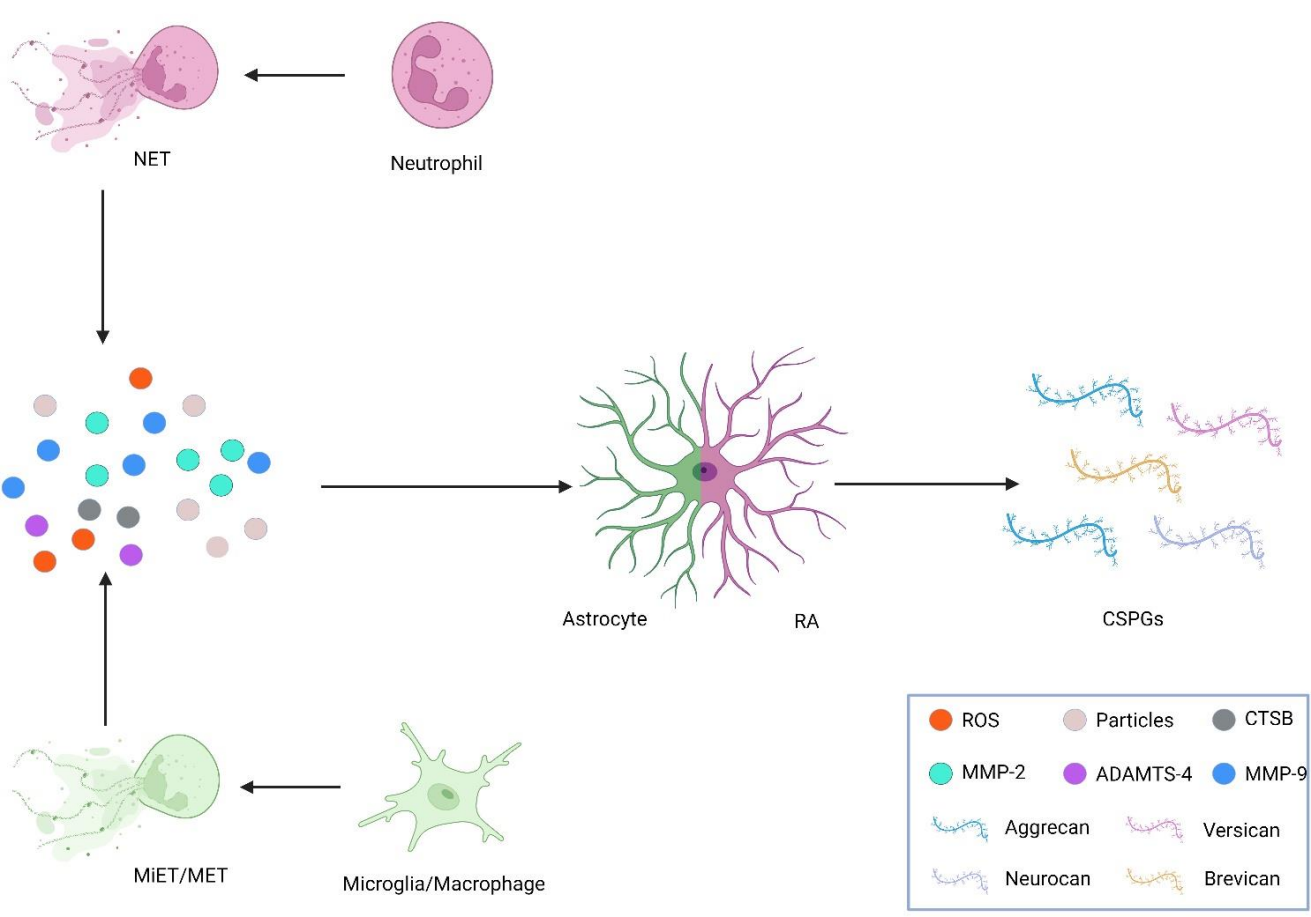
**ETs promote the formation of CSPGs after SCI. After SCI, neutrophils release all levels of granules and MMPs to promote the secretion of CSPGs from RA through the formation of NETs**. Microglia/macrophages may produce ET in SCI; ROS, reactive oxygen species; CTSB, Cathepsins B; ADAMTS-4, A disintegrin and metalloproteinase with thrombospondin motif-4.

The neutrophils and microglia/macrophages act as immune cells after SCI to regulate the inflammatory pathological process of secondary injury and promote glia scarring [2]. Recent studies have shown that neutrophils promoted the overexpression of CSPGs in SCI by degranulating and forming neutrophil extracellular traps (NETs) [5]. Moreover, macrophages/microglia also form macrophages extracellular traps (METs) or microglia extracellular traps (MiETs) [21, 22]. Furthermore, the signals released by these cells, which regulate the expression of CSPGs, converge on RAs and promote their overexpression through various mechanisms [11-14] (Fig. 1).

**Figure 2.**
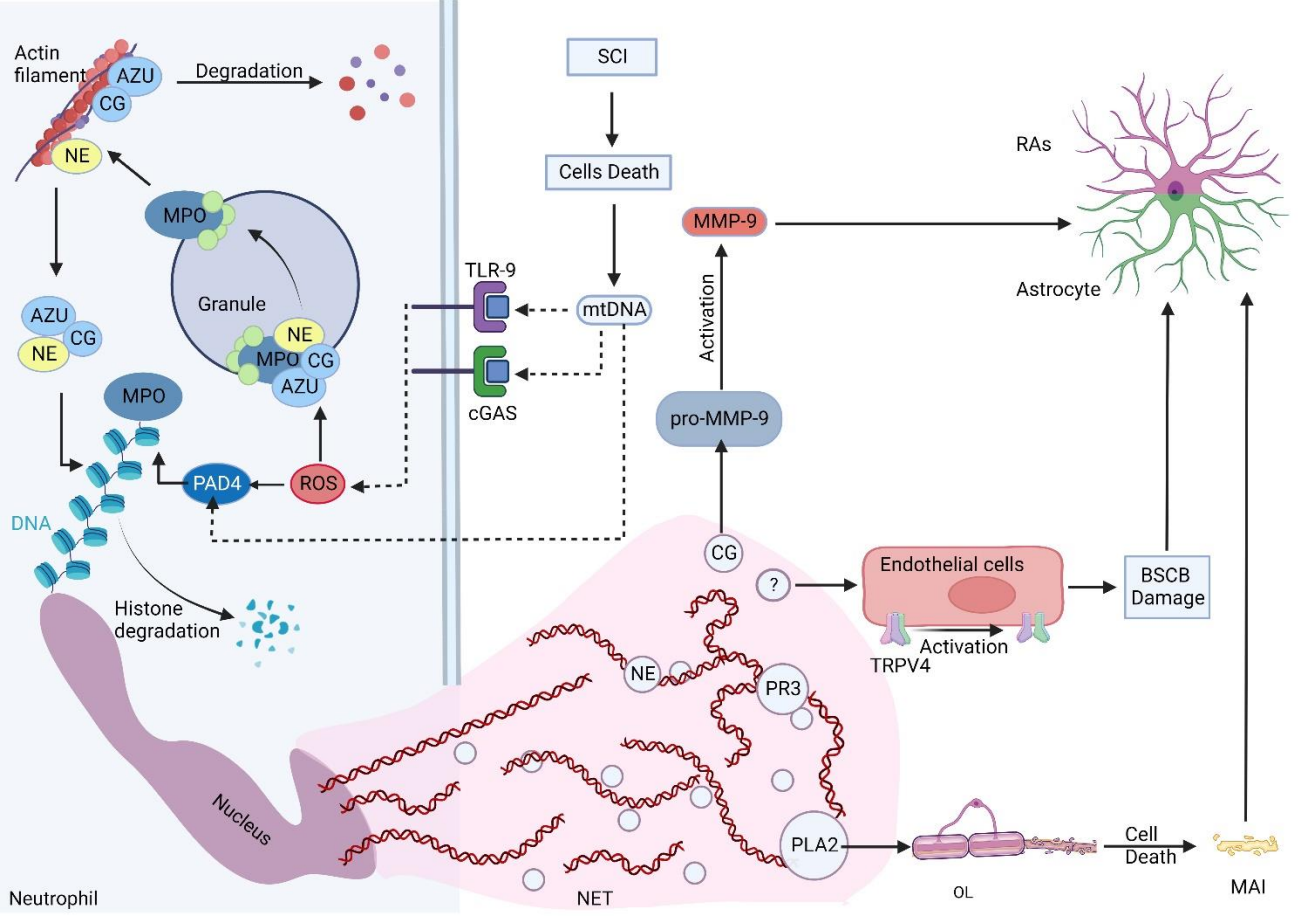
**Formation and role of NET after SCI**. SCI causes cell death, releasing cellular contents such as mtDNA. The mtDNA promotes the formation of ROS. MPO (Myeloperoxidase) mediated ROS oxidizes NE to degrade the actin cytoskeleton in the cytoplasm to block phagocytosis. NE then enters the nucleus, where it processes histones to condense chromatin. The MPO and PAD4 activation of citrullinated histones also promote chromatin de-concentration. It is released to form NET, which consists of DNA and particles of all levels. The CG particles activate pro-MMP-9. PLA2 triggers OL death and produces MAI. Some particles can also act as TRPV4 channels on the surface of BSCB endothelial cells, causing continuous BSCB leakage. Eventually, RAs are activated to promote the secretion of CSPGs. The dotted line in the figure indicates that the process has not been demonstrated in SCI. AZU, azurophilic granule; OL, oligodendrocytes.

### 2.1. Effects of ETs on the generation of CSPGs

The ETs, a DNA trap, are formed by the release of chromatin and granule proteins into the extracellular space by various immune cells, including neutrophils, mononuclear macrophages, and eosinophils, after a strong activation signal [23]. The NETs have been extensively studied in other diseases [24-26]. Neutrophils, as the first immune cells that reach the injury site in SCI, play a role in regulating inflammation and aggravating injury, thereby promoting secondary injury [27]. In recent years, NETs have been reported to have a unique role in aggravating inflammation and promoting astrocyte activation in SCI [5]. Moreover, recent research showed that microglia could also form ETs [21] (Fig. 2).

#### 2.1.1. Neutrophil extracellular traps

The NETs, first described in 2004 by Brinkmann, are reticular extracellular fibers capable of trapping bacteria [28]. It was believed that NETs were mainly activated by pathogens such as bacteria; however, recent studies found that they could also be activated and formed in aseptic inflammation, including SCI and traumatic brain injury (TBI), thereby playing an important role in promoting inflammation [5].

The exact mechanism of NETs formation in SCI has not been fully understood yet. In general, NETs are induced by the activation of peptidyl arginine deiminase 4 (PAD4), an enzyme that converts arginine on histones to citrulline to promote chromatin de-condensation [26]. Therefore, the activation of PAD4 is a key target for NETs formation. Moreover, neutrophil elastase (NE) also promotes chromatin de-condensation by degrading histones [26]. Liu et al. confirmed that mitochondrial DNA (mtDNA) could induce NETs formation in aseptic inflammation by establishing an acute peripheral tissue trauma model and skin injury model [29]. The mtDNA induces the increased production of NETs-associated proteins, such as Rac2 and PAD4, and also induces the release of reactive oxygen species (ROS), elastase, and histone 3 through cyclic GMP-AMP synthase (cGAS)-STING or Toll-like receptor 9 (TLR9)-mediated p38 mitogen-activated protein kinase (MAPK) and extracellular signal-regulated kinase 1/2 (ERK1/2) pathways [29] (Fig. 2). This model is similar to SCI, where the first hit causes sufficient cell death, thereby releasing package cellular contents and forming cell debris[30]. In conclusion, understanding the mechanism of NETs formation might provide targets and directions for therapy, such as PAD4 (see below).

The NETs can promote the activation of astrocytes through their components and secrete CSPGs. The NETs are mainly composed of granules, cytosolic proteins, proteases, histones, etc., which can indirectly or directly activate astrocyte [26]. For example, NE recruit neutrophils and other inflammatory cells to the site of injury by disrupting the substrates of the blood-spinal cord barrier (BSCB) and promoting the formation of glial scars [6]. Cathepsin G (CG) can convert pro-matrix metalloproteinase-9 (MMP-9), thereby activating MMP-9, which can further activate transforming growth factor-β (TGF-β) and promote glial scar [7, 10]. Phospholipase A2 (PLA2) in secondary particles increases the levels of excitatory glutamate, leading to neurotoxicity and demyelination by inducing oligodendrocyte death, which results in myelin debris [8]. In conclusion, the pathological results caused by these particles directly or indirectly activate RAs to secrete CSPGs. Moreover, Feng et al. found that NETs formation in SCI could promote neuroinflammation and mediate secondary BSCB destruction through the transient receptor potential vanilloid type 4 (TRPV4) channels [9]. However, the exact mechanism of activation is unknown yet. In conclusion, NETs formation exacerbates inflammation and further activates astrocytes; however, the specific activation mechanism is not understood yet.

#### 2.1.2. Microglial extracellular traps

Microglia can also form microglial extracellular traps (MiETs). Wang et al. demonstrated that interferon-γ (IFN-γ) could induce NADPH oxidase (NOX)-mediated MiETs formation in microglia, infected with *Listeria* [31]. This was the first time demonstrating that microglia could form ETs after bacterial infection. The main components include extracellular DNA (eDNA), MMP9 and MMP12, citrullinated histone 3 and peptidyl arginine deiminase 2 (PAD2), which are also vesicle components [31]. Its formation is dependent on cytosolic ROS and NO1X and is related to ERK. Another study showed that microglia could also produce MiETs under dopamine (DA) induction [32] , which was the first time, demonstrating that microglia produce ET under sterile conditions. Moreover, the dopamine-triggered ETs are independent of ROS formation and are not associated with cell death. Using flow cytometry and immunohistochemistry, Michel-Flutot et al. demonstrated that ETs were formed in microglia at the early stage of SCI and were concentrated around the lesion core [21]. However, the formation mechanism and role of MiETs have not been discovered yet, which could be important in SCI studies. The research studies on the role of NETs in SCI and MiETs in other CNS aseptic inflammation, speculate that MiETs might play a similar role as NETs in SCI [32]. In other words, MiETs might regulate and promote inflammation, and microglia might be concentrated in glial scars [33]. Therefore, in terms of the spatial distribution of microglia, MiETs might activate astrocytes, thereby promoting the deposition of CSPGs and forming a glial scar. Evans et al. showed that microglia cells were distributed in glial scars and macrophages in fibrous scars [34]. Moreover, macrophages and microglia in SCI are similar in function and morphology [35]. Furthermore, macrophages can also form ETs, called macrophage extracellular traps (METs) [36]. Therefore, studies should focus on understanding whether METs promote fibrous scarring and whether MiETs promote glial scarring after SCI.

In conclusion, the studies on ETs in SCI are in the initial phase. Neutrophils, macrophages, and microglia can all form ETs after SCI. However, their formation, function, and changes in time and space need to be studied in depth.

### 2.2. CSPGs are overexpressed by reactive astrocytes

**Figure 3.**
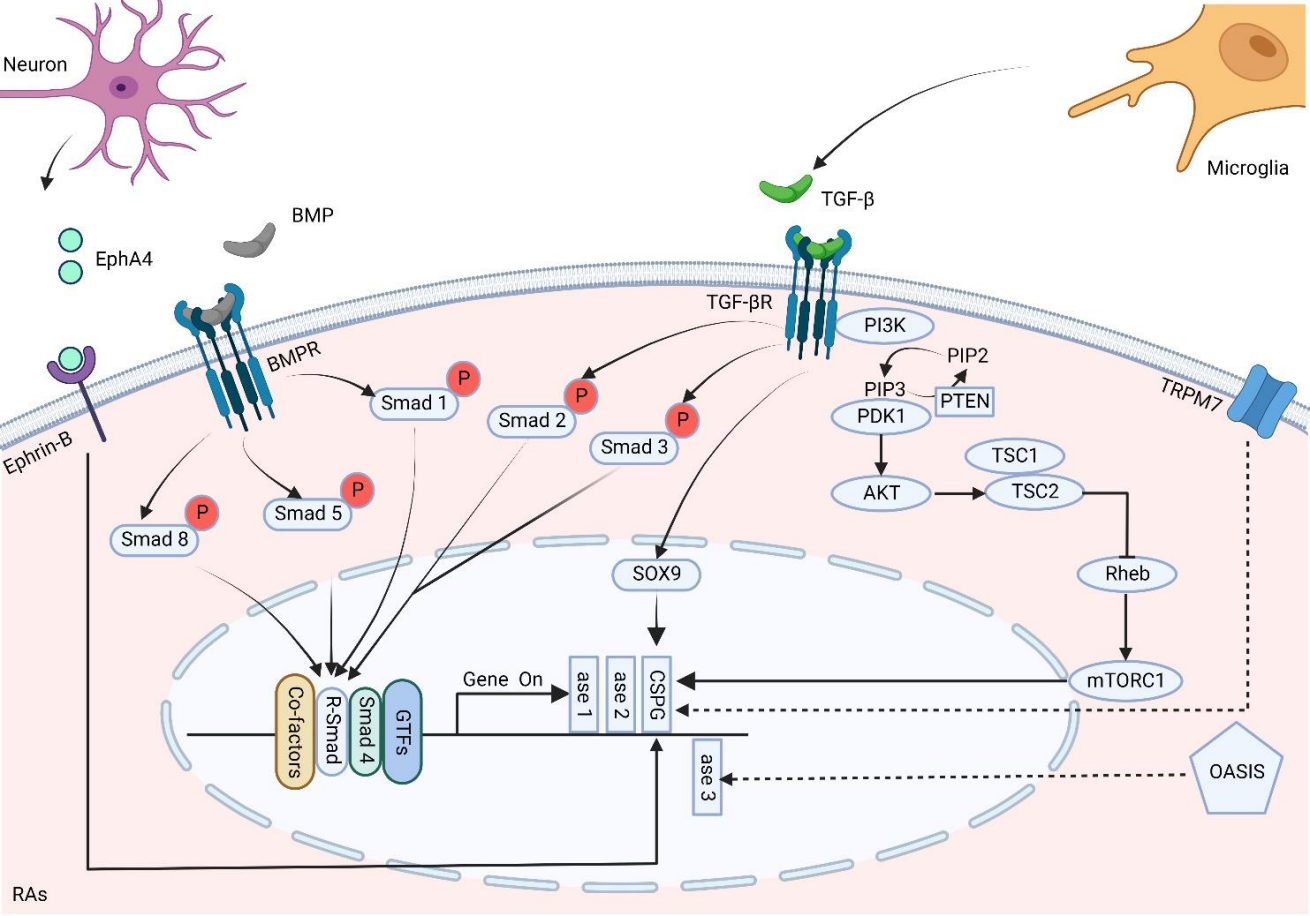
**CSPGs secretion mechanisms**. In SCI, TGF-β and BMP can overexpress CSPGs through SMAG-dependent pathway. TGF-β can be secreted by microglia and act on astrocytes through Smad2/Smad3 pathway to induce CSPGs overexpression. Smad3 is expressed with C4ST1 and 4-sulfated CSPGs. Smad2 mediates phosphacan and chondroitin synthase 1 expression. BMP induces CSPG overexpression through the Smad1/Smad5/Smad8 pathway. CSPGs overexpression can also be induced by TGF-β/SOX9 and PI3K/AKT/mTOR Smad independent pathways. In addition to the Smad pathway, EphA4 secreted by neurons interacts with Ephrin-B in astrocytes, which also causes CSPGs overexpression. In other diseases or injury sites, TRPM7 channels mediate CSPGs overexpression and OASIS induces increased C6ST1 expression. The dashed line in the figure indicates that this mechanism has not been explored in SCI. EphA4, Erythropoietin-producing hepatocyte A4; TRPM7, Transient receptor potential cation channel, subfamily M, member 7; PI3K, phosphoinositide 3 kinase; TSC1, Tuberous sclerosis 1; TSC2, Tuberous sclerosis 2; Rheb, Ras homolog enriched in brain; ase 1, C4ST1; ase2, chondroitin synthase 1; ase3, C6ST1; GTFs, general transcription factors.

The astrocytes perform several functions, including BSCB formation and "tetragonal synapses" formation with ECM molecules, under normal physiological conditions [37]. In SCI, astrocytes become reactive and form RAs under the stimulation of neutrophils, microglia/macrophages, necrotic cells, etc. [38]. RAs express several CSPGs through various mechanisms, including brevican, neurocan, versican, aggrecan, and mainly lectin-like CSPGs that form PNNs, after receiving signals from various cells and environmental cues [11-14] (Fig. 3).

#### 2.2.1. CSPGs generation by various reactive astrocytes subtypes

The CSPGs can be secreted through various signaling pathways in RAs, and their secretion is also diverse in different subtypes of RAs. The reason is that the "reactivity" of RAs in SCI depends on various factors, including the distance from the lesion core and intensity of the stimulus [3, 39, 40]. Moreover, Tamaru et al. found that in astrocytes, the expression of CSPGs decreased after 12 weeks of SCI (chronic phase), when RAs changed from scar-forming astrocytes (SAs) to chronic phase astrocytes (CAs) [41]. These reasons might lead to the different temporal and spatial effects of CSPGs secreted by RAs.

Currently, two subtypes of RAs can be distinguished [38], including proliferative, border forming RAs; and non-proliferative RAs. Non-proliferative RAs are located in spared but reactive neural tissue and interact with various cellular components, including neurons, synapses, and blood vessels [42].

The CSPGs that make up PNNs can be secreted by various cells, including neurons, microglia, astrocytes, etc. [15, 43, 44]. Among the subtypes of astrocytes, the CSPGs are secreted primarily by non-proliferative RAs located in spared but reactive neural tissue because they are not detected at the site of injury, where proliferating RAs are abundant. CSPGs act to limit synaptic plasticity rather than inhibit axon regeneration while surviving spared but reactive neural tissue [45-48]. Numerous studies suggested that the beneficial effects of blocking CSPGs receptors after incomplete SCI were related to the inhibition of synaptic plasticity by PNNs in spared but reactive neural tissue[3, 46, 49], with no axonal regeneration at the injury site. Moreover, studies also suggested that CSPGs inhibited axonal regeneration (see below).

In general, the above-mentioned differences in the distribution and function of CSPGs suggested that the proliferative RAs inhibited axonal regeneration, while non-proliferative RAs inhibited synaptic remodeling. However, in SCI, while there are several classification methods, the standard classification of astrocytes has not been determined yet [41]. The classification of RAs can be performed using single-cell sequencing and other technologies, to demonstrate the difference between CSPGs secreted by different RAs [50]. This classification might further help in exploring the differences in the secretion of CSPGs by different types of astrocytes in SCI as well as better targeting of different RAs to suppress the negative effects of CSPGs.

#### 2.2.2. Regulatory mechanisms of CSPGs secretion by RAs

After SCI, the ET- activated, RAs regulate the expression of CSPGs through multiple signaling pathways. The expression of CSPGs is mainly regulated by the Smad pathways, including Smad-dependent and Smad-independent pathways (Fig. 3).

##### 2.2.2.1. Smad-dependent pathways

The induction of Smad2/3 is different in the TGF-beta/Smad-dependent pathway. Among them, the expressions of 4-sulfated CSPGs and chondroitin-4-sulfotransferase-1 (C4ST1) are mediated by Smad3, while the expressions of phosphacan and chondroitin synthase 1 are mediated by Smad2 [11]. The bone morphogenetic protein (BMP), such as TGF-β, upregulates the expression level of CSPGs through the Smad-dependent pathway. However, the regulation of Smad by BMP4 and BMP7 is different from that of TGF-β. BMP mainly causes the phosphorylation of Smad1, Smad5, and Smad8 [12] and increases the expression level of core proteins in CSPGs in addition to the overexpression of CSPGs [12]. The Smad-dependent pathway is comprehensive to regulate the CSPGs expression, including the enzymes related to the formation of CSPG as well as the core protein of assembling polysaccharide peptide chains.

##### 2.2.2.2. Smad-independent pathway

Recent studies have shown that TGF-β could regulate the overexpression of CSPGs through a Smad-independent pathway. After SCI, microglia undergo M1 polarization and express TGF-β at 3 and 7 dpi [13]. The induction of CSPGs in RAs occurs via TGF-β/SOX9 signaling pathway [13]. Moreover, Jahan et al. found that TGF-β, which could activate the PI3K/Akt/mTOR signaling pathway through the Smad-independent pathway, also mediated this role [51]. Furthermore, Chen et al. demonstrated that the activation of PI3K/Akt/mTOR pathway in astrocytes could induce the secretion of CSPGs and promote the formation of glial scar at about 3 days after SCI [52]. The activation of the PI3K/Akt/mTOR pathway in neurons might have beneficial effects [1].

##### 2.2.2.3. Other signaling pathways

In addition to the Smad signaling pathway, the neuronal secreted EphA4, which acts on Ephrin-B in astrocytes, also increases the expression levels of CSPGs [53]. Moreover, Takazawa et al. found an increase in the old astrocyte-specifically induced substance (OASIS) after SCI was associated with an increase in RAs [14]. OASIS, a basic leucine zipper transcription factor of the cAMP response element, belongs to the binding/activating transcription factor family. It regulates the differentiation of neural precursor cells into astrocytes in the CNS [54]. This might explain the increase in CSPGs after SCI from the perspective of the number of RAs. Moreover, future studies should focus on the timing node selection to understand the PI3K/Akt/mTOR pathway for inhibiting the synthesis of CSPGs.

In the brain and other diseases, novel mechanisms, which promote the synthesis of CSPGs, have been discovered; however, they have not been demonstrated in SCI, yet. Okuda et al. found that OASIS was involved in the transcriptional regulation of the chondroitin 6-O-sulfotransferase 1 (C6ST1) gene in a rat cerebral cortex stab wound model and promoted the sulfation of CSPGs [55]. Moreover, in multiple sclerosis (MS), transient receptor potential cation channel, subfamily M, member 7 (TRPM7, a Ca2^+^-permeable nonselective cation channel) was highly expressed in RAs to mediate CSPGs production [56]. These findings should be further confirmed in SCI to explore a new mechanism of CSPGs secretion.

## 3. Roles of CSPGs Following SCI

In SCI, the inhibitory effects of CSPGs on axon regeneration have been extensively studied. The downstream pathway focused on in studies is Rho/ROCK, which eventually leads to growth cone collapse [2, 57]. Moreover, CSPGs are now found to have important and even beneficial roles in regulating inflammation and regulating cell migration and differentiation [17, 19, 20, 58].

### 3.1. Inhibitory effects of CSPGs on axon regeneration

After SCI, axonal regeneration requires cytoskeleton guidance in growth cones [59]. The proper assembly and disaggregation of microtubules are required for this process. Moreover, this process also requires the neuronal cell body to provide energy to the growth cone transport mitochondria in time [60]. The CSPGs phosphorylate different substrate proteins through the ROCK pathway, thereby mediating microtubule assembly and depolymerization failure [61]. Furthermore, recent studies have shown that CSPGs inhibited axon regeneration by inhibiting autophagic flux and mitochondrial trafficking in axons [62].

**Figure 4.**
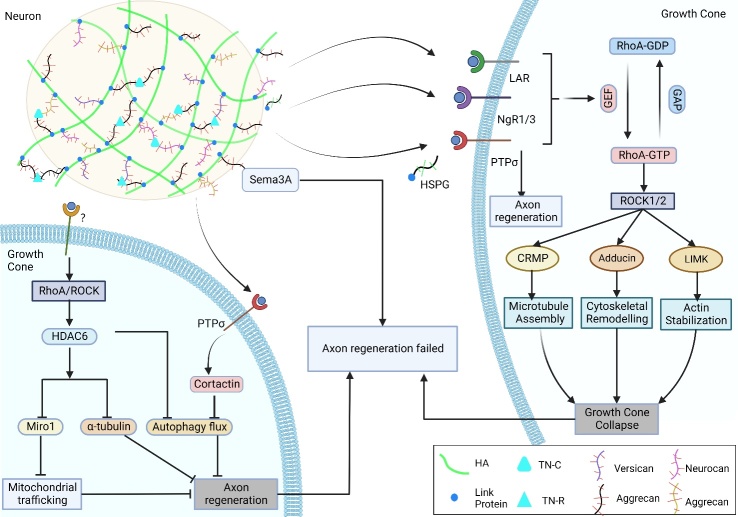
**CSPGs inhibit the mechanism of axon regeneration**. After SCI, CSPGs are secreted in large quantities to form PNN around neurons. Four main classes activate RhoA/ROCK pathway through interaction with growth cone receptors leading to growth cone collapse. On the one hand, CSPGs can mediate axonal mitochondrial transport, microtubule assembly, and autophagic flux leading to axon regeneration failure. HA, Hyaluronan. HSPG, Heparan sulfate proteoglycan.

#### 3.1.1. Microtubule dynamics

CSPGs act on receptors including, protein tyrosine phosphatase σ (PTPσ), phosphatase leukocyte common antigen-related (LAR), and nogo receptors 1 and 3 (NgR); the downstream pathways converge on the Rho/ROCK pathway and eventually lead to growth cone collapse [4] (Fig. 4). The downstream effector of Rho is ROCK, which phosphorylates CRMP (Collapsin Response Mediator Protein), LIM kinase (LIMK), and Adducin. The phosphorylation of these proteins leads to the disturbances in cytoskeletal dynamics [61]. Ultimately, all these events cause the cytoskeleton remodeling and growth cone collapse. CRMP directly regulates cytoskeletal dynamics and neurite elongation, and its phosphorylation leads to the loss of binding affinity for cytoskeletal proteins [63]. SCI induces the upregulation and phosphorylation of CRMP2/4 [64, 65]. Inhibition of CRMP2 phosphorylation stabilizes axonal microtubules, reduces inflammation, and inhibits scar formation [64, 65]. LIMK1 phosphorylates cofilin specifically at Ser-3, which hinders the actin-depolymerizing action of cofilin LIMK1 [66].

#### 3.1.2. Mitochondrial transportation

Mitochondria can produce energy at the growth cone (axon end), which is then transported from the cell body through microtubules [59]. Stabilizing microtubules after SCI can reduce axonal degeneration, prevent the formation of terminal spheres normally seen in nonpermissive environments, and support axonal regeneration [67]. Overexpression of CSPGs after SCI can mediate axonal mitochondrial transport and cytoskeletal motility (microtubule dynamics) to inhibit axonal regeneration [68]. HDAC6, a tubulin deacetylase localized in neuronal axons, can promote the deacetylation of substrates, including α-tubulin [69]. The axonal growth of CSPGs is promoted by the inhibition of HDAC6 [70]. Kalinski et al. showed that CSPGs could further activate HDAC6 by increasing intracellular Ca^2+^ through the ROCK pathway, which ultimately led to the reduced acetylation of miro1 [68]. Miro and Milton proteins can link mitochondria to motor proteins for mitochondrial axonal transport [71]. Therefore, CSPGs reduce mitochondrial transport and inhibit axonal regeneration through the above-mentioned processes.

#### 3.1.3. Autophagy flux

A novel downstream pathway that inhibits axon regeneration has been identified. The binding of CSPGs side chain CS to PTPRσ can dephosphorylate cortactin (here identified as a novel PTPRσ substrate) and disrupt autophagy flux at the autophagosome-lysosome fusion step, resulting in the formation of dystrophic growth cones [62]. Moreover, Zheng et al. found that HDAC6 inhibition in SCI could restore the autophagic flux and promote axonal regeneration [72]. Therefore, the control of autophagy flux is essential for axonal regeneration, not only as an important regulatory target in CSPGs, but also in the enhancement of endogenous neural forces. Tran et al. further proposed that CSPGs/ PTPσ-mediated autophagy flux adjustment after SCI could serve as a switch to mediate axonal growth or synaptogenesis [73].

In addition to the above-mentioned mechanisms of inhibiting axon regeneration, recent studies have reported that CSPGs/LAR/PTPσ-axis can suppress neuronal differentiation in part by blocking the Wnt/β-Catenin pathway [74]. However, the specific mechanism of action has not been explored yet. Therefore, several downstream pathways of CSPGs, which inhibit axon regeneration, need to be studied further.

### 3.2. Beneficial and harmful effects of CSPGs on inflammation

**Figure 5.**
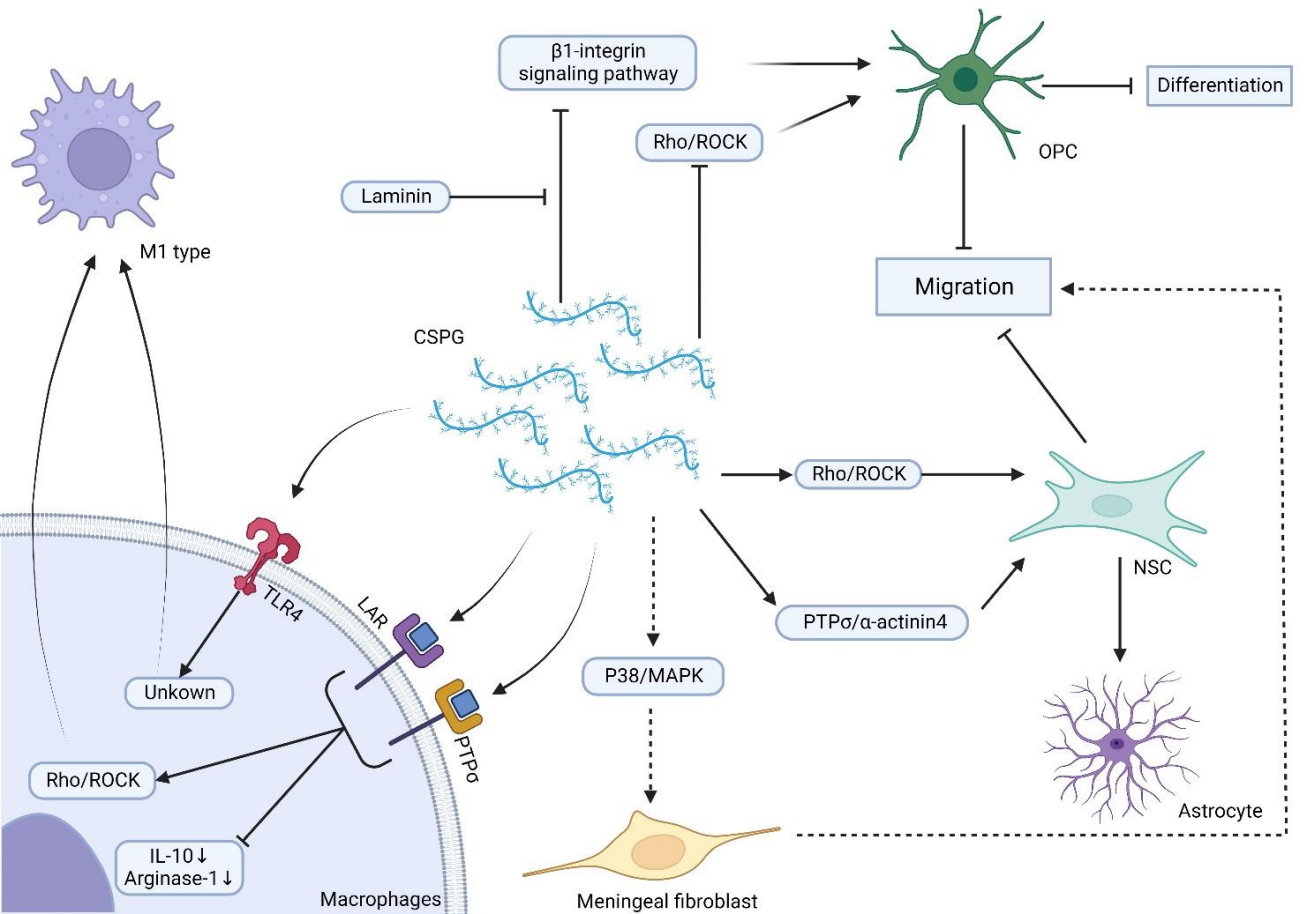
**CSPGs regulate inflammation and cell migration and differentiation mechanisms**. CSPGs promote and maintain the M1 phenotype by acting on TLR4, LAR, and PTP receptors in macrophages. The expression of IL-10 and ARG-1 also decreases. CSPGs inhibit OPC and NSC migration through different pathways and promote the migration of meningeal fibroblasts to the site of injury. Moreover, they inhibit OPC differentiation through different pathways and promote NSC differentiation into astrocytes. The dotted line indicates that it has not been explored in SCI. MAPK, mitogen-activated protein kinase.

CSPGs regulate inflammation by regulating the phenotypic transformation of microglia and macrophages [17, 58]. In a previous study, Bartus et al. delivered chondroitinase ABC (ChABC) in rat SCI using a lentiviral vector to massively digest CSPGs. They found increased expression levels of phagocytic macrophage marker CD68 after 3 days of injury followed by an increase in CD206 level after 2 weeks of injury, indicating that macrophage phenotype could be altered by large-scale digestion of CSPGs [17]. Dyck et al. further found that CSPGs reduced the synthesis of interleukin-10 (IL-10) and arginase-1 by acting on LAR and PTPσ, thereby promoting the transformation to or maintenance of the M1 phenotype in microglia and macrophages; moreover, its downstream action was partially achieved through the Rho/ROCK pathway [58] (Fig. 5). This was also found in a rat demyelinating disease model, which was treated with neuregulin-1. The rat model showed that the neuregulin-1 treatment significantly attenuated the upregulated expression of CSPGs in the extracellular matrix of demyelinating lesions and enhanced the production of IL-10 by immune cells [75]. IL-10 is an important cytokine involved in promoting M2 phenotypic transition [33]. Moreover, CSPGs not only attenuated IL-10 expression, which inhibited the M2 phenotype, but also promoted or maintained the M1 phenotype directly through Rho/ROCK pathway [58, 75]. Recent studies have shown that intracellular sigma peptide (ISP) and intracellular LAR peptide (ILP), supported by hydrogel, could effectively block the CSPG receptor and inhibit the formation of microglia's M1 phenotype, thereby promoting the formation of the M2 phenotype, improving the microenvironment, and reducing inflammation [76].

Recent studies have shown that CSPGs play a key role in preventing inflammation from regression [77, 78]. CSPGs blocked the conversion of pro-inflammatory immune cells to a repairing phenotype by acting on TLR4 on the surface of microglia or macrophages [77] (Fig. 5). Moreover, in MS and spontaneous meningitis disease models, Versican’s V1 subtype, accumulated in the perivascular cuff, could significantly upregulate proinflammatory cytokines and chemokines in macrophages [78].

However, the beneficial aspects of CSPGs should be noted in the early stage of SCI when considering the harmful aspects of CSPGs. Rolls et al. found that the inhibition of CSPGs immediately after injury decreased the expression levels of insulin-like growth factor 1 (IGF-1) production and increased those of tumor necrosis factor-α (TNF-α) in microglia and macrophages; however, the inhibition of CSPGs showed a beneficial effect if the inhibition was delayed for 2 days after injury [18]. The results showed that CSPG might play a critical role in the repair of the injured spinal cord as well as the recovery of motor function during the acute phase after the injury.

Briefly, in most wounds, macrophages tend to become M1 macrophages in the early stage in order to promote the removal of dead cell debris, while they tend to become M2 macrophages in the later stage to promote tissue repair [79]. However, in SCI, the M1 phenotype of microglia and macrophages is maintained for a long time [57]. Based on these observations, Bradbury et al. proposed the concept of "non-resolving pathology", suggesting that in the core of the lesion, microglia and macrophages remained in M1 type for a long time due to the failure of complete removal of necrotic debris in the core cells of the lesion [57]. Its phenotype did not undergo the transition from pro-inflammatory to anti-inflammatory associated with the next stage of wound healing in other organ tissues, thereby forming a cyst cavity. Later, a new development suggested that myelin debris was also involved in the pathological process [80]. After the phagocytosis of myelin debris, macrophages gradually form foamy cells and maintain the M1 phenotype for a long time [80]. Moreover, CSPGs are also actively involved in this pathological process, promoting and maintaining the M1 phenotype of microglia and macrophages for a long time [58, 75, 76]. Therefore, future studies should focus on targeting these inflammatory mechanisms regulated by CSPGs for treatment.

### 3.3. Differential regulation of CSPGs in the migration and differentiation of cells

Recent studies have found that overexpression of CSPGs could inhibit the migration of cells, including oligodendrocyte precursor cells (OPCs) and neural stem cells (NSCs) [19, 20]. However, it can promote the migration of meningeal fibroblasts to the site of injury [81] (Fig. 5). After SCI, the increase in CSPGs inhibits the migration and differentiation of OPCs, possibly by activating the inhibitory effects of Rho kinase production. A decade ago, Siebert and Pendleton demonstrated both *in vitro* and *in vivo* that CSPGs could inhibit the differentiation of OPC into oligodendrocytes as well as its migration to the injury site after SCI; however, the inhibitory effects of CSPGs on these cells could be reversed by treating with ChABC [19, 20]. Moreover, Kuboyama et al. demonstrated that CSPGs were broad universal regulators of OPCs differentiation [82]. Karus et al. further demonstrated that the PTPσ/ROCK pathway mediated the inhibitory effects [83]. MMP-2 might be a downstream molecular target regulated by PTPσ [84]. The upregulation of MMP-2 enables OPCs to digest, migrate and differentiate through CSPGs [84]. In MS, the elevation of Versica in CSPGs members at the lesion was found to directly impede the migration and differentiation of OPCs, which indirectly inhibited remyelination by enhancing local Th17 cytotoxic neuroinflammation [85]. Sun et al. measured the effects of CSPGs on the proliferation, migration and differentiation of OPCs *in vitro*, and found that this might be related to the effects of CSPGs on OPCs as well as the downregulation of β1-integrin, indicating that CSPGs might be competitively inhibited by β1-integrin signaling pathway [86]. Moreover, they also found that the inhibitory effects of CSPGs were counteracted by laminin [86]. Regardless of the downstream pathways, CSPGs could inhibit the migration and differentiation of OPCs into OLs, promote the PTPσ /ROCK/MMP-2 pathway, and inhibit β1-integrin signaling [83, 84, 86].

In addition to inhibiting the migration and differentiation of OPCs, CSPGs can also inhibit the migration and differentiation of NSCs, mediated through various pathways. Galindo et al. showed that CSPGs could inhibit the migration of NSCs *in vitro* by activating ROCK [87]. Moreover, Zhong et al. found that CSPGs could inhibit the migration of neural stem-progenitor cells (NSPCs) and promote them into astrocytes through the PTPσ/α-actinin4 signaling pathway [88]. Furthermore, in oxygen-glucose deprivation conditions, CSPGs could promote the migration of meningeal fibroblasts via the p38 MAPK signaling pathway [81]. These findings suggested that CSPGs were biased towards scarring and were not conducive to nerve regeneration. However, these studies are rare in SCI, and the mechanistic role of these CSPGs in cell migration might be promising new targets for promoting nerve regeneration.

## 4. Treatment Strategies

The SCI, treatment based on CSPGs is mainly carried out in three perspectives: removing CSPGs, preventing CSPGs formation, and manipulating CSPGs receptors and signaling [2, 57]. The current review article focuses on proposing therapeutic strategies for some of the new targets mentioned above.

### 4.1. Inhibition of ETs formation to reduce RAs activation

The inflammation caused by the microbes, including *Candida albicans*, *Mycobacterium bovis*, etc., might further lead to the spread of bacteria; therefore, the inhibition of NET requires careful consideration [26]. However, in aseptic inflammation such as SCI, inhibiting the formation of NET or promoting the degradation of NET can alleviate inflammation [89]. Currently, PAD4 inhibitors, including Cl-amidine, are mainly used to inhibit NET formation [90-93]. Moreover, DNase1 content can be increased to promote NET degradation to inhibit the negative effects of NET [94]. However, preventing the early recruitment of neutrophils can aggravate SCI injury [95]. It is suggested that the NET formation can promote scarring, thereby limiting further inflammation. Further studies are needed to refine the spatiotemporal changes of NET in SCI, precisely select the time of action, inhibit its effect, and achieve the best therapeutic effects.

### 4.2. Inhibition of CSPGs synthesis

After SCI, it is best to inhibit the synthesis of CSPGs in the first place [2, 57, 96]. CSPGs can be removed or disrupted by numerous methods, including inhibition of their synthesis, enzymatic degradation, antibody neutralization, and pharmacological targeting of effector molecules. However, these methods should be applied in the acute phase of the injury, thereby limiting their clinical use. They can directly inhibit pathways that stimulate CSPGs production; for instance, Pan et al. showed that Schwann cell-derived exosomes (SCDEs) reduced the deposition of CSPGs by increasing TLR2 expression on astrocytes via the NF-кB/PI3K signaling pathway (Table 1) [97]. Alternatively, neuregulin-1 can mediate the reduction of inhibitory CSPGs through ErbB2 tyrosine phosphorylation in the ErbB2/3 heterodimer complex [98]. It can also be performed at the genetic level, such as knocking down *C4ST1* gene [99], a key enzyme for CSPGs synthesis, or knocking down the *Sox9* gene associated with the inhibition of axon regeneration [100]. It is also possible to knock down the genes that express CSPGs-acting receptors; however, Rodemer et al showed that knocking down the *PTPσ* gene expression after complete spinal cord transection caused reduced regeneration of axons [101], suggesting that PTPσ has other beneficial roles. Finally, CSPG synthesis can be reduced by directly reducing SAs recruitment. Tamaru et al. blocked β1 integrin and inhibited SAs recruitment from surrounding astrocytes in the chronic SCI phase without altering the SAs phenotype. The results showed a reduction in glial scar volume and promotion of axon regeneration, thereby suggesting its potential as a possible treatment approach for chronic SCI patients [41].

**Table 1:** Effects of different treatments on CSPGs.

Treat	Changes of CSPGs	Signaling pathway	In vivo/ vitro	Ref.
**Mechanical stimuli**	Downregulation	Unknown	In vitro	[111]
**Inhibit autophagy**	Upregulation	TGF-β-mediated	In vitro	[112]
**AP1, AP2**	Downregulation	Unknown	In vitro	[113]
**Paclitaxel**	Downregulation	Unknown	vivo	[114]
**SCDEs**	Downregulation	NF-κB/PI3K	In vivo	[97]
**Fluorosamine**	Downregulation	Unknown	In vitro	[115]
**MiR-379-5p**	Downregulation	Unknown	In vivo	[116]
**IFN-γ**	Downregulation	Unknown	In vivo	[117]
**HGF**	Downregulation	Unknown	In vitro	[118]
**2-AG**	Downregulation	Unknown	In vitro	[119]

AP1: andrographolide (AP1); SCDEs: Schwann cell-derived exosomes; HGF: Hepatocyte growth factor; 2-AG: 2-arachidonoylglycerol

### 4.3. Inhibition of CSPGs receptors

The CSPGs can also be inhibited by blocking the CSPGs’ receptors [2, 57, 76]. This therapeutic strategy can be applied in both acute and chronic phases after injury. Researchers designed an ISP, an innovative peptidomimetic of PTPσ, that changes even after the growth cone has entered a terminal spherical state, thereby allowing progress in CSPGs-laden fields [4]. Moreover, the inhibition of the LAR receptor by ILP promoted axonal growth on CSPGs substrates *in vitro* [58]. Sun et al. used the hydrogel carrier to transport ISP and ILP into the body, which effectively improved the pro-inflammatory microenvironment, promoted axon regeneration, and avoided the spread of inflammation caused by the large area digestion of CSPGs [76]. Therefore, as compared to ChABC therapy, the targeted blocking of CSPGs’ receptors might achieve three beneficial effects, including reduced inflammation, weakened effects of CSPGs blocking axon regeneration, and avoiding the complete digestion of scar tissue, leading to the spread of inflammation.

### 4.4. Inhibition of the downstream targets of CSPGs

HDAC6 is an important target, regulating the axonal autophagic flux, mitochondrial transport, and microtubule movement. CSPGs enhance the effects of HDAC6 through the ROCK pathway [68, 72, 102]. Therefore, the effects of regulating energy supply and cytoskeletal movement in axon regeneration can be achieved simultaneously by inhibiting HDAC6. Currently, HDAC6 inhibitors mainly include TSA, tubacin, tubastatin A, ACY-1215 (ricolinostat), ACY-241 (citarinostat), etc. [72, 102, 103]. In addition to inhibiting HDAC6, these inhibitors can also enhance the expression of αTAT1, which has an opposite effect on HDAC6, such as lentiviral-mediated αTAT1 overexpression [104]. However, the use of HDAC6 inhibitors in SCI has been less explored. HDAC6 might be a potential therapeutic target of CSPGs by mediating multiple downstream mechanisms to inhibit axon regeneration.

### 4.5. Enzymatic digestion of CSPGs

Sometimes, it is necessary to remove the synthesized CSPGs in SCI when it is too late to inhibit CSPGs synthesis. The most classic enzyme for CSPGs digestion is ChABC; a recent study showed that the multiple intraparenchymal Chase injections under the lesion, which targeted the spinal cord circuit controlling hand function, significantly improved the hand function of ChABC-treated monkeys [105]. Moreover, better therapeutic effects could be achieved by combining the enzymatic hydrolysis of CSPGs with the promotion of synaptic recombination. Suzuki et al. designed CPTX, a synaptic organizer protein, to promote synaptic and motor function recovery after SCI. They showed that in a contusion SCI model, the combination of CPTX with ChABC could achieve the best therapeutic effect [106]. CSPGs can also be removed by utilizing endogenous regulators and specifically targeting the core proteins following SCI. The most widely studied is ADAMTS4, a proteolytic enzyme, that catalyzes the core CSPGs protein. A recent study showed that the ADAMTS4 degraded CSPGs by using adenoid-associated virus vector gene therapy [107]. The CSPGs core protein can also be cleaved using MMP; however, its effect is not as strong as that of ADAMST4 [108]. The above-mentioned approaches for SCI treatment by targeting CSPGs are only part of the treatment. However, in actual treatment, they can be combined with other treatment strategies, such as improvements in gut microbiota and inhibition formation of fibrous scar [109, 110].

## 5. Conclusions

CSPGs are re-emerging in SCI with a new look. At the early stage of SCI, NETs and MiETs can activate astrocytes, promote the production of CSPGs, and form glial scars. The studies on ETs in SCI and other CNS diseases have just begun. NETs, METs, and MiETs play unique roles in SCI. CSPGs are used to focus on inhibiting axon regeneration in SCI; however, they play an important role in regulating inflammation as well as cell migration and differentiation. Given the wide distribution of CSPGs after SCI, this regulatory effect is very important. Therefore, it is necessary and effective to target CSPGs for SCI treatment. The adverse effects of CSPGs can be eliminated at multiple levels, including the inhibition of CSPGs production, blocking of the CSPGs receptor, inhibition of CSPGs downstream pathway, and finally the degradation of CSPGs. However, it is important to note that CSPGs might be beneficial in early stage of SCI. In general, CSPGs should be re-examined from generation to function and from temporal variation to spatial distribution.
